# Biological in-vivo measurement of dose distribution in patients' lymphocytes by gamma-H2AX immunofluorescence staining: 3D conformal- vs. step-and-shoot IMRT of the prostate gland

**DOI:** 10.1186/1748-717X-6-62

**Published:** 2011-06-07

**Authors:** Felix Zwicker, Benedict Swartman, Florian Sterzing, Gerald Major, Klaus-Josef Weber, Peter E Huber, Christian Thieke, Jürgen Debus, Klaus Herfarth

**Affiliations:** 1 Department of Radiation Oncology, University of Heidelberg, Heidelberg, Germany; 2 Clinical Cooperation Unit Radiation Oncology, DKFZ, Heidelberg, Germany

## Abstract

**Background:**

Different radiation-techniques in treating local staged prostate cancer differ in their dose- distribution. Physical phantom measurements indicate that for 3D, less healthy tissue is exposed to a relatively higher dose compared to SSIMRT. The purpose is to substantiate a dose distribution in lymphocytes *in-vivo *and to discuss the possibility of comparing it to the physical model of total body dose distribution.

**Methods:**

For each technique (3D and SSIMRT), blood was taken from 20 patients before and 10 min after their first fraction of radiotherapy. The isolated leukocytes were fixed 2 hours after radiation. DNA double-strand breaks (DSB) in lymphocytes' nuclei were stained immunocytochemically using the gamma-H2AX protein. Gamma-H2AX foci inside each nucleus were counted in 300 irradiated as well as 50 non-irradiated lymphocytes per patient. In addition, lymphocytes of 5 volunteer subjects were irradiated externally at different doses and processed under same conditions as the patients' lymphocytes in order to generate a calibration-line. This calibration-line assigns dose-value to mean number of gamma-H2AX foci/ nucleus. So the dose distributions in patients' lymphocytes were determined regarding to the gamma-H2AX foci distribution. With this information a cumulative dose-lymphocyte-histogram (DLH) was generated. Visualized distribution of gamma-H2AX foci, correspondingly dose per nucleus, was compared to the technical dose-volume-histogram (DVH), related to the whole body-volume.

**Results:**

Measured *in-vivo *(DLH) and according to the physical treatment-planning (DVH), more lymphocytes resulted with low-dose exposure (< 20% of the applied dose) and significantly fewer lymphocytes with middle-dose exposure (30%-60%) during Step-and-Shoot-IMRT, compared to conventional 3D conformal radiotherapy. The high-dose exposure (> 80%) was equal in both radiation techniques. The mean number of gamma-H2AX foci per lymphocyte was 0.49 (3D) and 0.47 (SSIMRT) without significant difference.

**Conclusions:**

*In-vivo *measurement of the dose distribution within patients' lymphocytes can be performed by detecting gamma-H2AX foci. In case of 3D and SSIMRT, the results of this method correlate with the physical calculated total body dose-distribution, but cannot be interpreted unrestrictedly due to the blood circulation. One possible application of the present method could be in radiation-protection for *in-vivo *dose estimation after accidental exposure to radiation.

## Introduction

In radiotherapy, high doses have to be delivered to the tumour. However, sparing of healthy tissue and organs at risk is essential. Variations can be made by increasing the number of radiation beams, which leads to differences in dose distribution between two radiation-techniques: the three dimensional conformal (3D) and the Step-and-shoot-IMRT (SSIMRT). According to the number of beams, the irradiated volume as well as the dose-distribution can change. Smaller volume has to be compensated by higher dose to reach the prescribed target dose inside the tumor. In our prostate radiotherapy protocol, the 3D-conformal therapy contains 4 beams, whereas in SSIMRT, dose is distributed within 7-9 beams. The distribution of low doses is broader in a larger volume in SSIMRT.

Using the gamma-H2AX stain to detect DNA-double strand breaks (DSB) in human lymphocytes is known as an established method [1]. Localized near or at irradiation induced DSB, the H2AX histones are phosphorylated sensitively to provide signalling within the DNA DSB-repair. As one DSB represents one gamma-H2AX focus, it is possible to visualize DSB immunocytochemically using a fluorescence microscope [2,3]. The number of foci can be used as a reliable parameter to estimate the delivered dose, since it increases linearly with the induction of DSB [4]. These cellular responses are equally efficient at different doses. But there is an evidence, that the activation of DNA-repair needs a certain level of DNA damage; approximate 1 mGy [5].

It has to be considered, that gamma-H2AX foci are an indirect marker and that equalization with the exact number of DSB, especially after repair, is currently a debate [6,7].

Lymphocytes can easily be taken from the patient's peripheral vein and, due to the described method, used as biological dosimeters. The focus of the study lies on the dose distribution within the lymphocytes measured indirectly by gamma-H2AX foci in patients undergoing radiotherapy in the prostate region. Whether the results can serve as a surrogate for dose distribution in the irradiated body volume and therefore for a new method of biological dosimetry must be discussed critically. Limitations have to be taken into consideration, e. g. circulation of the lymphocytes in the body during irradiation [4].

The purpose of this study is to visualize the cellular effect of ionizing radiation during prostate cancer treatment, by evaluating the dose-distribution using the gamma-H2AX immunodetection in human lymphocytes. If possible, we want to verify the differences in dose distribution between 3D conformal and SSIMRT with biological methods.

## Material and methods

### Patients and Irradiation

Individuals analyzed in this study were all males, with a median age of 71.4 years (range 51.1 - 83.6), and had an indication for irradiation of the prostate region. This selection was made, because the DNA damage level depends on the anatomic region [8]. Exclusion criteria were a prior radiation in the patients' medical history (so no exposition in advance could interfere with the test) or the additional radiation of lymphatic regions of the pelvis. For either treatment method (3D, SSIMRT), 20 patients were recruited. All patients gave their informed consent. The study was approved by the ethics committee of the University hospital of Heidelberg. The patients' treatment was not influenced by the study and indications for the different modalities were made clinically. Further patient data comparing 3D with SSIMRT is shown in Table 1. The body volume was calculated by the formula as it is published for male patients [9]:

**Table 1:** Data of prostate cancer patients, which were treated by 3D (n = 20) or SSIMRT (n = 20)

	3D		SSIMRT	
	**median**	**range**	**median**	**range**

**body volume (l)**	86.0	69.88 - 154.8	85.46	53.75 - 103.2

**planned target volume (cm^3^)**	132.0	83.0 - 319.2	181.0	71.8 - 337.1

**age (years)**	69.7	51.1 - 83.6	71.6	65.4 - 81.9

The radiation was performed by a department's linear accelerator (Oncor, Siemens). Table 2 contents the technical parameters of the two irradiation modalities. To calibrate absolute doses to the investigated number of gamma-H2AX foci, blood of 5 volunteers was irradiated *in-vitro *for 3 independent measurements on different days. Utilization of volunteers was necessary because of intended test repetition, not suitable for patients. Inter-individual differences were considered by investigating 5 subjects. The venous blood was irradiated with doses of 0.02, 0.1, 0.5, 1 and 2 Gy by the same linear accelerator used for the irradiations of the patients. The object-to-focus distance was 1.58 m, the radiation field 10 × 5 cm. Radiation absorbing plates were stacked to a 20 cm tower to allow very low dosage; so the beam on time reaches the operating range of the linear accelerator after the stabilization phase. By varying the time of radiation, different doses were applied. Dose was measured by relative online dosimetry (DIN 6800-2) by using an ionization chamber (thimble 0,3 cm^3^, PTW, Freiburg, Germany).

**Table 2:** Technical data: 3D vs.IMRT

	3D	SSIMRT
**beams**	4	7-9

**boost**	sequential	integrated

**SD (Gy)**	2	2.17

**CD (Gy)**	72	76

**fractions**	36	35

**energy (MV)**	18	6

**dose output (MU/min)**	300	300

**mean beam-on time (min)**	1.29	6.16

**mean table time (min)**	11.5	16.3

SD = Single fraction dose, CD = Cumulative dose, MV = Megavolts, MU = Monitor units.

### Lymphocyte separation and immunofluorescence analysis

7.5 ml of patient's blood were taken from a peripheral vein 10 min after the first fraction of the treatment. The blood circulation was given 10 minutes after fraction to mix the radiated lymphocytes with the rest that hadn't been exposed to radiation. Non-exposed controls were also taken before radiation.

The protocol of staining gamma-H2AX by indirect immunofluorescence is published in many papers and its purpose for detecting DNA DSB validated [10, 11, 12, 13, 14 and 15]. Lymphocytes were separated from the blood by layering 5 ml of heparinized, venous blood onto 3 ml of Ficoll and centrifuging at 2300 rpm for 20 min at 37°C. The lymphocytes were washed in 6 ml of PBS-buffer and centrifuged at 1500 rpm for 10 min (37°C). After aspirating the buffer, the cell-pellet was re-suspended in a 1:15 ratio. 200 μl of this suspension, containing about 300,000 lymphocytes, were spread onto a clean slide by means of the Cytospine Centrifuge at 22 rpm for 4 min (room temperature). Fixating the lymphocytes took 10 minutes (room temperature) in fixation buffer (3% paraformaldehyde, 2% sucrose in PBS). For all experiments, this step was performed 2 hours after finishing radiation to allow comparability between the samples. In order to allow the antibodies getting inside the nucleus, the cells were permeabilized for 4 min at 4°C (permeabilisation buffer: 20 mM HEPES (pH 7.4), 50 mM NaCl, 3 mM MgCl_2_, 300 mM sucrose, and 0.5% Triton X-100). Samples were incubated with anti-gamma-H2AX antibody (Anti-Phospho-Histone-gamma-H2AX Monoclonal IgG-mouse-Antibody (# 05-636), Upstate, Charlottesville, VA) at a 1:500 dilution for 1 h, washed in PBS 4 times, and incubated with the secondary antibody (Fluoresceiniso-thiocyanat(FITC)-conjugate, Alexa Fluor 488 Goat-anti-mouse-IgG-conjugate, Molecular Probes, Eugene, OR) at a dilution of 1:200 for 0.5 h. Both incubations took place at 37°C. Cells were then washed in PBS four times at room temperature and mounted by using VECTASHIELD mounting medium including the nucleus stain DAPI (Vector Laboratories). Thus, the gamma-H2AX foci could be correlated with the nuclei.

The slides were viewed with an × 100 objective (fluorescence-microscope Laborlux S, Leica Microsystems CMS GmbH, Wetzlar, Germany). The spots inside the nucleus were counted by eye because of the possibility to focus manually through the whole nucleus by microscope to detect each focus in the 3D-room. All experiments were counted by one and the same, trained person. For each of the samples, 300 lymphocytes were analyzed within the patient samples with its heterogeneous dose-distribution. All nuclei were morphologically considered by eye (cell form and size) to be properly shaped and in G0/1-phase with haploid chromosome-set.

Due to their homogenous radiation, *in-vitro *samples and controls were investigated by counting 50 cells each experiment and measuring point. Three independent experiments were done.

### Data and statistical analysis

For every patient, gamma-H2AX foci of the lymphocytes were counted. For every count of gamma-H2AX foci per nucleus the averaged relative number of cells was calculated from 20 patients each group (3D and SSIMRT).

The calibration curve involved five subjects irradiated at six different doses in three independent measurements. Background foci levels were subtracted. As the relationship between dose application and irradiation induced gamma-H2AX foci formation is linear [4], a linear regression curve was generated, which implies the following general formula:

(Y = number of gamma-H2AX foci per nucleus, × = dose in Gy, m = gradient)

This linear regression curve was used to calculate an equivalent dose for every count of irradiation induced gamma-H2AX foci per nucleus in patients' lymphocytes. Background foci were subtracted again (controls before irradiation). In addition, the values of gamma-H2AX foci were converted into relative doses, whereas 100% corresponds to the given dose of 2.0 Gy (3D) and accordingly 2.17 Gy (SSIMRT). The calibration concerns only the single lymphocyte, irrespectively body site or blood flow.

In a further integral diagram, the relative number of lymphocytes with gamma-H2AX foci was plotted against the relative applied dose in %. Each point shows the cumulative number of lymphocytes exposed to a certain dose, or more. This visualization of distribution of radiated lymphocytes was defined as dose-lymphocyte-histogram (DLH).

The original dose-volume-histograms (DVH) were modified in order to compare them to our generated DLHs: in general, the volume percentage in the DVH refers to the contoured volume of the CT-scanned part of the body (aortic bifurcation to the thigh). The data was standardized by referring it to the individual's total body volume, allowing interpretation equivalent to the DLH. With the rule of proportion the values of the contoured volumes can transferred to values of total body volumes.

Formula:

The statistics were done by Sigma Plot 10.0^®^. The level of significance was set at p < 0.05 using a Student's t-test.

## Results

### *In-vitro *measurements for calibration curve

**Figure 1:**
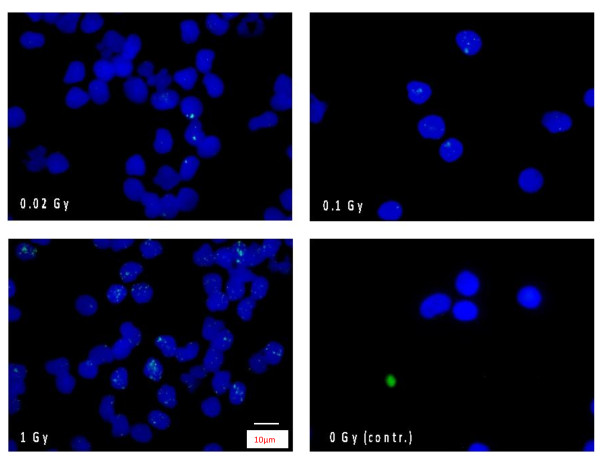
**Merged DAPI and gamma-H2AX stains in human blood lymphocytes**. Number of phosphorylated H2AX-foci corresponds with the dose. Different doses are shown: 0.02, 0.1, 1 Gy and the non irradiated sample. Irradiation was performed homogeneously *in-vitro *on a linear accelerator (Oncor, Siemens).

**Figure 2:**
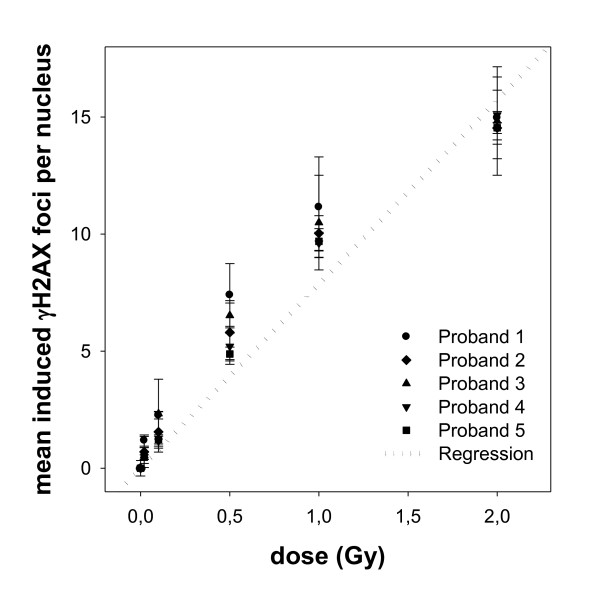
**The calibration curve was set-up by irradiating blood samples of five volunteers and is used to correlate the delivered dose with the mean number of induced gamma-H2AX foci per nucleus, scored 2 hours after irradiation**. Background foci levels were subtracted. Lymphocytes were irradiated ex vivo at six different doses (0 - 2Gy) in three independent measurements each (standard deviations are shown).

The relation of dose and mean number of gamma-H2AX foci per nucleus (see also Figure 1) of all 5 subjects' lymphocytes follows the same characteristic without significant differences (p > 0.05), which confirms the absence of inter-individual differences [16]. The estimated regression line is used as a calibration curve (Figure 2) and its formula is:

(Y = number of gamma-H2AX foci per nucleus, × = dose in Gy)

For example, 0.5 Gy correlates with a mean number of gamma-H2AX foci per nucleus of 4.9, 1 Gy with 8.6 and 2 Gy with 16 foci, 2 hours after irradiation.

### *In-vivo *measurements of patients' lymphocytes

**Figure 3:**
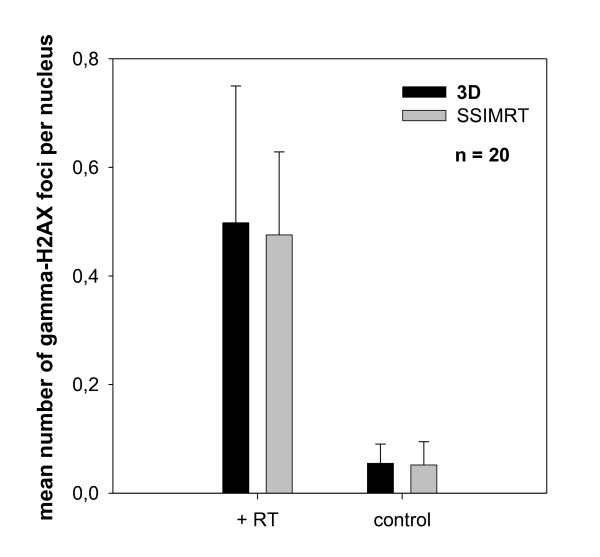
**The average of mean number of gamma-H2AX foci per nucleus in irradiated lymphocytes and negative controls of 20 patients per group is shown (3D and SSIMRT)**. Standard errors are shown. All patients were irradiated upon their prostate region, whereas venous blood was taken before (control) and 10 minutes after their first irradiation fraction. Lymphocytes were fixed 2 h after the end of the irradiation. In the negative control 50 lymphocytes were analyzed per patient, while in the irradiated samples, 300 lymphocytes were analyzed per patient.

Related to investigated lymphocytes of 20 patients per group the mean number of gamma-H2AX foci per nucleus is 0.49 (3D) and 0.47 (SSIMRT) in the irradiated samples (Figure 3), while the non-irradiated control marks 0.06 (3D) and 0.05 (SSIMRT). The number of foci in the samples after irradiates were for all the patients larger than the number of foci in the non-irradiated control samples. The bars show significant difference between irradiated samples and the control (p ≤ 0.05). The mean number of gamma-H2AX foci in both radiation modalities is the same (p > 0.05).

### Dose-lymphocyte histogram (DLH)

**Figure 4:**
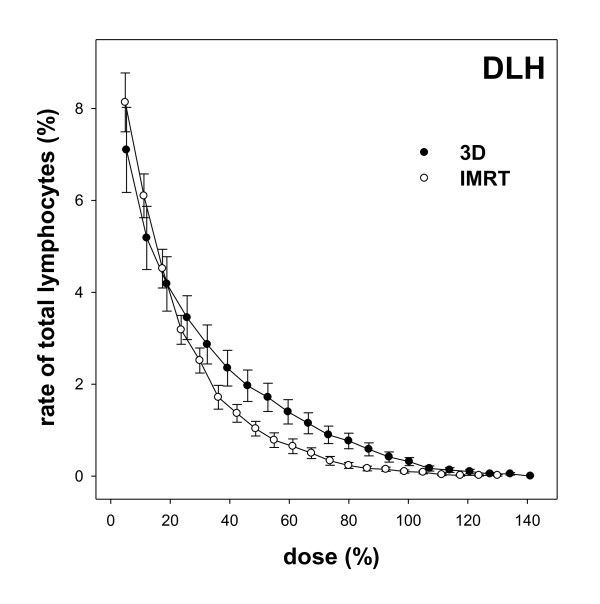
**Dose-lymphocyte-histogram (DLH)**. In this integral histogram, data of 20 patients per group (3D and SSIMRT) are summarized in two curves. Standard errors are shown. The dose initially was correlated with each number of γH2AX foci. Background foci levels were subtracted. Referring to a previously generated calibration line (Figure 2), the count of γH2AX foci leads to the equivalent delivered dose for each lymphocyte. Each point contains the mean relative sum of lymphocytes with at least the shown relative dose (≥ x). 100% dose is equivalent to 2 Gy for 3D and 2.17 Gy for SSIMRT. This causes the slight shift between the points of the curves.

The DLH is a cumulative histogram; each point shows the cumulated number of lymphocytes that has been exposed to a certain dose, or more (Figure 4). Background foci-levels have been subtracted, since they were also subtracted in the calibration line. The curves cross at about 20% of the described dose, while the SSIMRT curve lies above the 3D curve at lower doses and below it at higher doses. The significant difference is obvious between 40% and 90% of the delivered dose: here, the SSIMRT curve lies significantly below the 3D curve (p ≤ 0.05). There is no difference in relative number of lymphocytes, which get more than 95% of the applied dose. The percentage of lymphocytes exposed to more than 50% of the prescribed dose is 1.8% in 3D technique, compared to 0.9% in SSIMRT.

### Dose-volume histogram (DVH)

**Figure 5:**
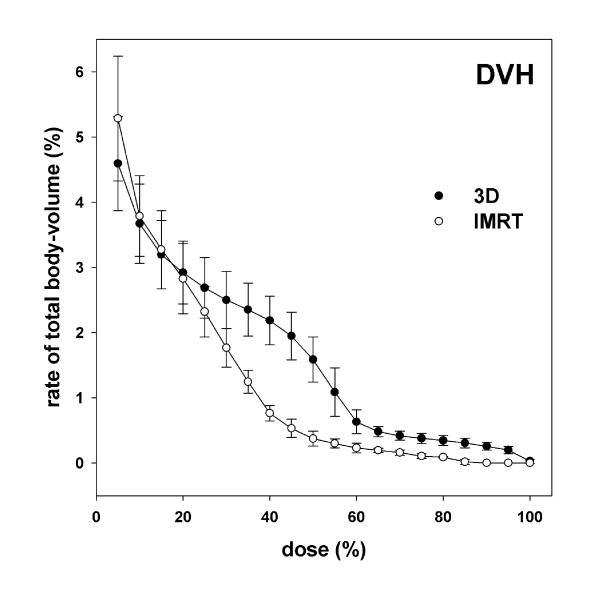
**Dose-volume-histogram (DVH)**. Origin for this diagram was the irradiation planning data of a smaller selected group of 3D- and SSIMRT-prostate-patients from the main pool. Each curve of this integral histogram contains 5 patients, each point contains the volume irradiated with at least the shown relative dose (≥ x). Standard deviations are shown.

The curves' crossing point in the DVH takes place at just below 20% of the described dose, whereas the SSIMRT lies above the 3D at 0%-20% and significantly (p ≤ 0.05) below it between 30%-95% (Figure 5). The percentage of volume exposed to more than 50% of the prescribed dose is 1.7% in 3D technique, compared to 0.4% in SSIMRT.

## Discussion

Lymphocytes of patients receiving irradiation for the treatment of prostate cancer have been analyzed by scoring gamma-H2AX foci. A distribution of delivered dose to the lymphocytes is shown and visualized in the graphics above. Similarity between DLH (dose-lymphocyte-histogram) and DVH (dose-volume-histogram) has been found. The biological measurement on behalf of the human lymphocytes corresponds to the distribution calculated by the physicists: more low-dose-delivery is observed for the SSIMRT compared to the 3D. At the same time, a lower distribution of 30%-90% of the applied dose can be reported for the SSIMRT.

The advantage of this method is an easy and fast access to the required material without any massive medical interventions. The method allows an in vivo estimation respectively proof of the dose distribution calculated by the therapy planning system.

The challenge is that every patient has to be irradiated at a comparable volume and same site of the body. Attention also has to be paid to the repair kinetics and withdraw of gamma-H2AX foci, which make it necessary to stop cell metabolism after a certain duration post irradiation. Due to this context, we fixed all cells 2 h after irradiation (*in-vivo *and *in-vitro*) to allow comparability between the samples.

However, the determination of the probability of lymphocytes' presence in the body tissue is difficult, due to the lymphocytes' kinetics (circulation in the blood vessels), migration and adhesion to the vessel wall. These circumstances have been described by Sak et al. in detail [4]. It has to be considered, that lymphocytes in in-field capillaries move slower and receive more dose, than fast moving lymphocytes in larger vessels. Sak et al. described differences in mean numbers of gamma-H2AX foci in lymphocytes depending on irradiated target sites, e.g. brain and thorax. In our study, target site was no variable parameter, since we compared 3D and SSIMRT only in prostate cancer treatment.

The SSIMRT's beam-on-time differed from the 3D's by a factor 5 (Table 2). Assuming a blood circulation time of one minute, this fact causes inaccuracy while measuring the actual dose distribution. On the other hand, table time in both modalities differs by factor 1.4. During 11.5 vs. 16.3 min of table time, lymphocytes in both groups have the chance of being radiated more than one time. The cumulative formation of gamma-H2AX foci can lead to a false high result in evaluating dose distribution. In order to attempt a correction towards real dose distribution in SSIMRT, one would expect even less cells exposed to higher levels of dose. This correction would amplify the differences between 3D and SSIMRT, which again correspond with the physical model.

Statement implying an absolute dose in Gy used for dosimetry, cannot be recommended without doubts, due to the following issues: in the DLH (Figure 4) higher lymphocyte-percentages are plotted, compared to the DVH (Figure 5). The DLH shows a radiation dose of 5% in 7-9% of lymphocytes (DLH), whereas only about 5% of the body volume receives the same dose (DVH). Doses of above 100% can be observed in the DLH, too. This phenomenon can be explained by the possibility of repeated dose exposure of some lymphocytes as explained above.

The linear correspondence between induction of γH2AX foci and the delivered dose has already been verified and practiced especially for low doses [4,17]. Exceptions from this rule are described and due to different irradiation conditions or different kinds of ionizing irradiation [18].

The visualization, which is shown for computed tomography examinations of different sites (1), was now extended to the doses of one fraction of radiotherapy for different techniques.

Flow cytometry has also been performed in order to measure delivered dose by γH2AX stain [16], however, in our case it didn't seem appropriate: The intensity of the gamma-H2AX foci varied and could have led to errors while measuring the background level of fluorescence. In our opinion, a concrete number of foci per nucleus is needed to compare dose distribution exactly.

Jucha et al. evaluated 2-dimentional pictures of the stained lymphocytes using special software [19], but we set great store by being able to zoom through the slide under the microscope and looking at the complete 3-dimentional nucleus in order to detect every gamma-H2AX foci. For this reason in our experiments foci were counted manually by eye with a fluorescence-microscope.

By creating a dose-lymphocyte histogram (DLH), the gamma-H2AX staining method allows the estimation of the dose distribution after irradiation. One possible application of the present method could also be in radiation-protection for *in-vivo *dosimetry after accidental exposure to radiation. In case of accidental irradiation, background foci level cannot be determined and therefore cannot be subtracted in the DLH. In this situation background foci level should also not be subtracted in the calibration line. In this manner the error due to background foci level can be reduced, however individual differences of background foci levels remain unconsidered. Another possibility to deal with this limitation is to take blood for background foci level examination several weeks after the exposure, when the circulating lymphocytes have been substituted naturally.

## Conclusion

Measurement of γH2AX foci in patients' lymphocytes after prostate irradiation has been performed and dose distribution within the lymphocytes shown. SSIMRT delivers more doses below 20% and less between 30%-90% than 3D. This new biological *in-vivo *method confirmed the reduction of medium-dose-exposure for normal tissue by SSIMRT. The relation between actually distributed dose (DVH) and distribution of gamma-H2AX foci in lymphocytes (DLH) shows similarity but cannot be interpreted unrestrictedly due to the blood circulation.

## Conflict of Interest

The authors declare that they have no competing interests.

## Authors' contributions

FZ conceived of the study, carried out patients' mentoring and experiments and drafted the manuscript. BS carried out the the gamma H2AX experiments and helped to draft the manuscript. CT helped to draft the manuscript. FS, GM, KW, PH and JD participated importantly in the conception of the study and provided informatics and support with statistics for data analysis. KH participated importantly in the conception and design and helped to draft the manuscript. All authors read and approved the final manuscript.
